# After a Decade of Therapy Revolution in Cutaneous Melanoma—Perspectives on Emerging Treatment Strategies

**DOI:** 10.32604/or.2026.078650

**Published:** 2026-05-21

**Authors:** Sebastian A. Wohlfeil, Jochen S. Utikal

**Affiliations:** 1 Skin Cancer Unit, German Cancer Research Center (DKFZ), Heidelberg, Germany; 2 Department of Dermatology, Venereology and Allergology, University Medical Center Mannheim, Ruprecht-Karl University of Heidelberg, Mannheim, Germany; 3 DKFZ Hector Cancer Institute, University Medical Center Mannheim, Mannheim, Germany

**Keywords:** Melanoma, immune checkpoint inhibition, neoadjuvant therapy, cancer vaccines, bispecific antibodies

## Abstract

Over the past decade, the therapeutic paradigm of cutaneous melanoma has been transformed strongly, driven by advances in immuno-oncology and precision medicine. Building on the success of immune checkpoint blockade and targeted therapy, new treatment strategies now aim to improve efficacy, overcome resistance, and prolong the durability of responses. Clinical trials on neoadjuvant therapy supporting its clinical use are presented. Furthermore, the latest progress in combinatorial immune checkpoint inhibition such as dual anti-LAG-3 or anti-TIGIT with anti-PD-1 blockade, next-generation bispecific antibody development, mRNA-based vaccines in clinical practice, and intralesional therapies are summarized. Additionally, it outlines the growing significance of novel cellular approaches, such as adoptive cell therapy with tumor-infiltrating lymphocytes (TILs) or engineered T cells. By integrating insights from recent clinical and translational research, the review highlights promising therapeutic avenues and with treatment sequencing and biomarker research, it outlines key challenges for future melanoma management. This review aims to summarize selected ongoing clinical studies and outline prospective directions in systemic melanoma therapy.

## 1 Introduction

**Figure 1:**
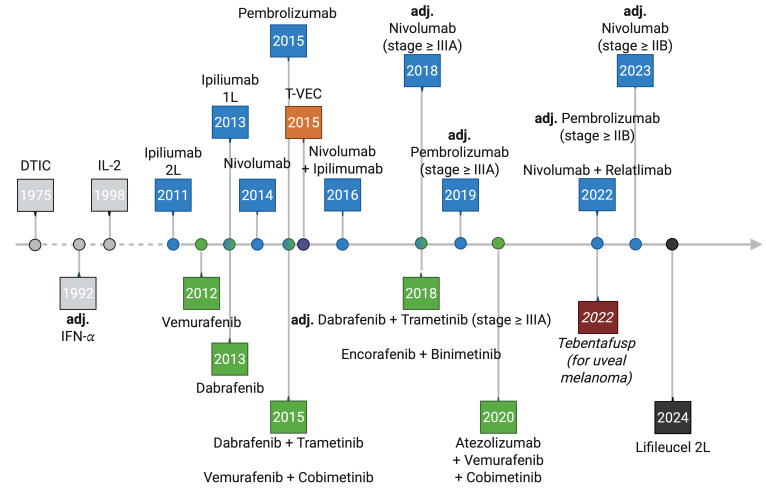
Timeline of cutaneous melanoma treatment evolution. The timeline shows the FDA’s approval of the current standard of care therapies for melanoma. If therapies were approved as an adjuvant modality, this is explicitly stated; otherwise, they are palliative treatments for unresectable disease. Old therapeutic agents are displayed in grey color, immune checkpoint inhibition (ICI) in blue, targeted therapy (TT) in green, Talimogen-Laherparepvec (T-VEC) in orange, Tebentafusp in red and Lifileucel in black. Adj. = adjuvant; 1 L = first-line; 2 L = second-line. Created in BioRender.

Cutaneous melanoma (CM) is a malignant tumor arising from melanocytes of the skin. It most frequently metastasizes to the skin, lymph nodes, lung, liver and brain [1]. In the last decade, the prognosis of metastatic CM has improved considerably following the introduction of immune checkpoint inhibitors (ICI) targeting programmed cell death protein 1 (PD-1) and cytotoxic T-lymphocyte-associated protein 4 (CTLA-4), as well as targeted therapies (TT) with BRAF and MEK inhibitors for *BRAF*-mutated CM [2,3] (Fig. 1; Supplementary Table S1). Additionally, the combination of anti-lymphocyte activation gene 3 (LAG-3) and PD-1 antibodies is used for unresectable CM as it has demonstrated superiority over PD-1 monotherapy [4]. The combination of relatlimab, an anti-LAG-3 inhibitor, and nivolumab, an anti-PD-1 inhibitor, demonstrates strong clinical responses, with a 3-year overall survival (OS) rate of 54.6% and an acceptable safety profile characterized by grade 3 or 4 adverse events in 22% of patients [5]. Furthermore, talimogene laherparepvec (T-VEC), a genetically modified herpes simplex virus type 1 producing granulocyte-macrophage colony-stimulating factor, is approved as oncolytic viral therapy in unresectable CM with clinically detectable lymph node or cutaneous metastases without distant metastases [6].

For certain cases of non-*BRAF*-mutated melanoma, additional targeted inhibitors can be used based on the tumor’s specific mutational profile. *NRAS* mutations are the second most common in CM [7]. In later line therapies, *NRAS*-mutated CM may be treated with a MEK inhibitor as monotherapy [8]. In certain *KIT*-mutant melanoma subsets, imatinib and other receptor tyrosine kinase inhibitors may be beneficial [9]. For CM with mutations in the *neurofibromin 1* tumor suppressor gene, recent experimental data suggest a treatment approach with epidermal growth factor receptor (EGFR) inhibitors [10].

To reduce the risk of distant metastatic disease, both ICI and TT are administered as adjuvant treatment in patients with stage III CM with locoregional metastases and in those with stage IV disease following complete resection of metastatic lesions, as these approaches significantly prolong relapse-free survival (RFS) and distant metastasis-free survival (DMFS) [11,12,13]. With respect to OS, adjuvant TT demonstrates a trend toward improved outcomes in the overall population, reaching statistical significance in patients with the *BRAF V600E* mutation, but not in those harboring the *BRAF V600K* mutation [11]. Interestingly, real-world data demonstrate a favorable PFS of *BRAF*-mutated stage III CM with adjuvant TT in comparison to ICI in German, Dutch and Polish cohorts [14,15,16]. However, this does not reflect on patients who started adjuvant therapy because of a disease relapse [17]. Dual ICI with nivolumab plus relatlimab failed to demonstrate any benefit over nivolumab monotherapy in the adjuvant RELATIVITY-098 trial [18]. This lack of efficacy, in contrast to the positive results observed in the palliative RELATIVITY-047 study, has been attributed to lower levels of LAG-3-expressing CD4^+^ and CD8^+^ T cells in the adjuvant cohort, where macroscopic tumors were absent. Adjuvant PD-1 blockade has additionally been approved for patients with high-risk stage IIB and IIC cutaneous melanoma without involvement of the nodal basin, on the basis of demonstrated improvements in DMFS and RFS [19,20].

However, in stage IV CM, approximately half of the patients receiving ICI and two-thirds of those treated with TT ultimately succumb to the disease due to primary or acquired resistance [2,3]. In stage III disease, approximately one-third of patients develop distant metastases within the first four years after receiving adjuvant anti-PD-1 inhibition [21,22]. Real-world data from German centers demonstrate a recurrence rate of 53% for adjuvant PD-1-treated patients vs. 44.5% for adjuvant TT at four years [23]. With regard to TT, acquired resistance to TT is common within the first year [24]. Secondary mutations or alternative splicing may promote reactivation of mitogen-activated protein kinase (MAPK) signaling or bypass it via alternative pathways, such as the phosphoinositide 3-kinase–mammalian target of rapamycin (PI3K-mTOR) signaling pathway [24]. In ICI, approximately half of patients do not respond to checkpoint inhibition in the initial phase (primary resistance) or during treatment (acquired resistance) [25]. Primary resistance is mediated by multiple parameters of melanoma cells or their microenvironment, such as low programmed cell death ligand 1 (PD-L1) expression, low numbers of infiltrating T cells, low tumor mutational burden, or the presence of immunosuppressive cells, e.g., myeloid-derived suppressor cells (MDSCs) [26,27,28,29]. Acquired resistance to ICI can be promoted by tumor-intrinsic effects, for example disturbed antigen presentation by mutations in the beta-2-microglobulin gene, or tumor-extrinsic regulators, such as T cell exhaustion or immunosuppressive properties of the tumor microenvironment [30,31,32]. Interestingly, there is also cross-resistance between TT and ICI, as resistance to BRAF and MEK inhibitors confers resistance to ICI via an immunosuppressive microenvironment characterized by a lack of CD103^+^ dendritic cells [33].

Furthermore, metastases to specific sites, particularly the brain and liver, are associated with reduced responses to these therapeutic modalities [34,35,36,37].

**Figure 2:**
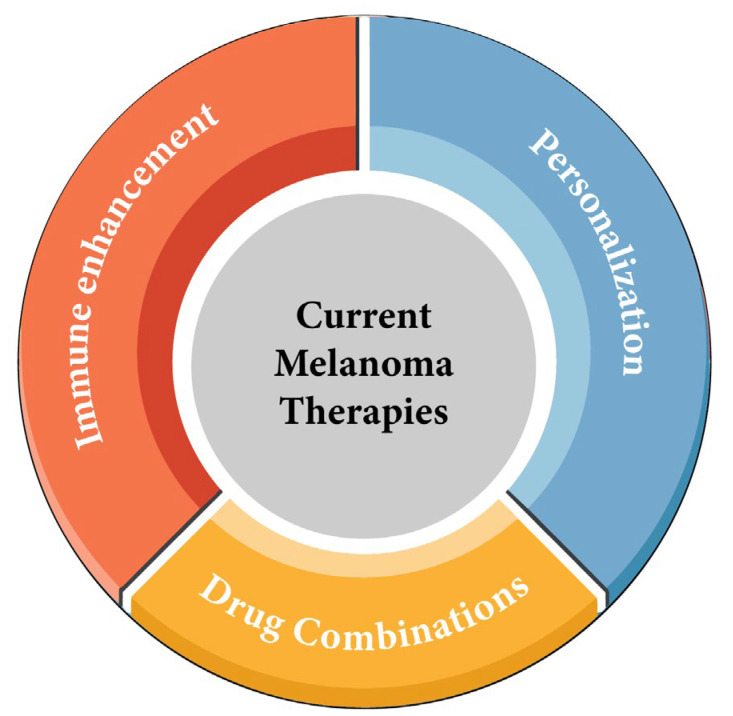
Key themes in cutting-edge melanoma treatment. 1. Personalization: therapy tailored to genetic and immune profiles (e.g., neoantigen vaccines, tumor infiltrating leukocytes (TILs), blockade of specific signaling pathways); 2. Drug Combinations: targeting of multiple immune targets to overcome therapy resistances; 3. Immune enhancement: engineered immune cells or viral therapies to generate precise, potent anti-tumor responses.

Consequently, novel therapeutic strategies are urgently needed to improve clinical outcomes and overcome resistance mechanisms in cutaneous melanoma (Fig. 2). This review aims to summarize selected ongoing clinical studies and outline prospective directions in systemic melanoma therapy (Supplementary Table S2).

## 2 Neoadjuvant Treatments

In patients with resectable, clinically detected CM, stage ≥ IIIB respectively, neoadjuvant treatment strategies represent an attractive approach to further enhance pathological response and significantly improve event-free survival (EFS) in comparison to currently approved adjuvant therapies. In principle, this treatment regimen exploits increased antigen presentation and broader immune cell activation in the presence of tumor cells [38].

The feasibility and efficacy of neoadjuvant ICI with nivolumab, an anti-PD-1 antibody, plus ipilimumab, an anti-CTLA-4 antibody, were demonstrated in the OPACIN trial led by Blank and colleagues [39]. Its successor, the OPACIN-neo study, refined this approach by identifying an optimized dosing regimen that preserves high antitumor activity while reducing immune-related toxicity [40,41]. The PRADO trial, an expansion cohort of OPACIN-neo, evaluated a response-adapted strategy in which the extent of surgery and the use of adjuvant therapy were tailored according to the pathological response in the index lymph node, thereby aiming to reduce treatment-related morbidity [42]. The phase III landmark NADINA trial demonstrated that neoadjuvant dual ICI significantly improves EFS to 83.7% compared with 57.2% for adjuvant PD-1 inhibition alone, while also increasing the rates of major pathological response in the index lymph nodes [43]. Furthermore, patients with a pathological complete response (pCR) maintain durable clinical benefit for twelve months even after the end of adjuvant therapy [43]. Another pivotal study, SWOG S1801, showed that administering three neoadjuvant doses of the PD-1 inhibitor pembrolizumab followed by consecutive adjuvant therapy significantly improves event free survival. In the standard adjuvant treatment group, it was 49%, while it was 72% in the neoadjuvant setting [44]. Regarding dual LAG-3 and PD-1 inhibition, data from smaller patient cohorts in a phase II trial indicated that neoadjuvant ICI with relatlimab plus nivolumab induces durable clinical responses, too [45]. In addition, the combination of fianlimab and cemiplimab is currently being investigated in a neoadjuvant phase II trial (NCT06190951). Most recently, the neoadjuvant application of tobemstomig, a bispecific antibody (bsAb) against PD-1 and LAG-3, which was investigated in a phase Ib/II trial (NCT05116202), showed promising results with a pCR in 80% of patients with tobemstomig compared to 77.3% with nivolumab and ipilimumab [46]. In contrast to neoadjuvant ICI, neoadjuvant TT with BRAF and MEK inhibitors, while achieving solid initial clinical responses, is generally associated with less durable benefit and increased risk of recurrence, thereby positioning immunotherapy as the preferred neoadjuvant approach in this setting [47].

Real-world evidence from Swedish and Swiss cohorts further supports the use of neoadjuvant therapies, confirming clinical response rates and EFS outcomes observed in clinical trials [48,49]. These advances are now incorporated into updated National Comprehensive Cancer Network (NCCN) and European Society For Medical Oncology (ESMO) melanoma guidelines and have led to the approval of perioperative therapy in several countries, including Australia and the Netherlands, although such approval is still pending in several countries, such as the USA and Germany [50,51].

## 3 Novel ICIs

Given the robust and durable clinical activity of dual checkpoint blockade with nivolumab plus ipilimumab, including an OS rate of 43% at the final 10-year analysis of the CheckMate-067 trial, the efficacy benchmark for future clinical trials in CM is high [25]. This is also substantiated by real-world data from Denmark and the Netherlands [52,53]. However, this benefit is accompanied by substantial toxicity, with grade 3 or 4 treatment-related adverse events reported in 62.6% of patients receiving nivolumab plus ipilimumab. Therefore, patients’ comorbidities must always be considered when tailoring individualized therapeutic concepts. Especially, this is important for frail patients, who may be offered anti-PD-1 monotherapy [54] or dual ICI with nivolumab and relatlimab instead of dual ICI with nivolumab and ipilimumab.

The development of the LAG-3-inhibitor relatlimab demonstrated that other immune checkpoints than CTLA-4 are worth targeting with an acceptable toxicity profile and is approved for advanced CM. Currently, results from ongoing clinical trials, conducted by a competitor, evaluating fianlimab, a novel anti-LAG-3 antibody, plus cemiplimab, another PD-1-inhibitor, in both palliative (NCT05352672) and adjuvant settings (NCT05608291) are highly anticipated.

T cell immunoglobin and ITIM domain (TIGIT) has been discussed as promising immune checkpoint and analyzed in experimental and clinical studies. TIGIT competes against the activating receptor CD226 and acts as inhibitory receptor on immune cells promoting T cell suppression [55]. The combination of TIGIT and PD-1 significantly enhanced the proliferation and degranulation of CD8^+^ tumor-infiltrating lymphocytes (TILs) isolated from melanoma patients *in vitro*, indicating an increased anti-tumor immune response [56]. However, clinical trials showed mixed results. The efficacy and safety of various anti-TIGIT inhibitors are currently analyzed in phase II basket trials with advanced solid tumors, including melanoma (NCT05060432). Besides, phase II clinical trials are currently investigating anti-TIGIT antibodies in combination with PD-1 inhibition in CM patients who have demonstrated resistance to upfront PD-1 monotherapy (NCT05483400, NCT05130177). Furthermore, in a phase I clinical trial, involving patients with solid tumors, predominantly non-small cell lung cancer, the combination of vibostolimab, an anti-TIGIT antibody, and pembrolizumab demonstrated satisfactory antitumor activity with a manageable safety profile [57]. However, the adjuvant phase III KeyVibe-010 trial (NCT05665595) in patients with resected high-risk CM stage IIB-IV was discontinued, as the combination of vibostolimab and pembrolizumab showed significantly increased toxicity as compared to PD-1 monotherapy.

Another inhibitory immune checkpoint receptor, T cell immunoglobulin and mucin-domain containing-3 (TIM-3), represents a compelling therapeutic target, as its co-blockade with PD-1 inhibition has led to improved anti-tumoral responses [58,59]. Early phase I and II clinical trials in patients with solid tumors, including CM, have reported an acceptable safety profile, along with preliminary clinical responses [60,61]. Furthermore, the phase I AMBER study in patients with treatment-naive or PD-1-refractory advanced cutaneous melanoma demonstrated promising treatment responses [62].

Early preclinical studies have also explored additional immune checkpoint receptors, including V-domain Ig suppressor of T cell activation (VISTA) [63,64] and B and T lymphocyte attenuator (BTLA), and it will be intriguing to observe their potential translation into clinical trials.

## 4 Cancer Vaccines

### 4.1 mRNA Vaccines

The extensive development of mRNA-based vaccine platforms during the COVID-19 pandemic has reignited interest and accelerated progress in tumor vaccine research. Also in CM, cancer vaccines are a highly promising immunostimulatory approach. These are injected intravenously or subcutaneously. While peptide vaccines can be processed and presented on major histocompatibility complex class I and II directly after internalization, mRNA vaccines must first be translated in the cytoplasm. Typically, these cancer vaccines express tumor-specific markers, such as (neo)antigens, leading to an enhanced antigen presentation and anti-tumor response. The following principal strategies are currently being pursued in clinical trials against CM: fixed mRNA vaccines incorporating predefined antigenic targets (for example BTN111 [NCT04526899]), individualized mRNA vaccines designed around patient-specific neoantigen repertoires (for example V-940 [NCT05933577, NCT06961006], autogene cevumeran [NCT03815058]), and peptide vaccines (for example IO102-IO103 [NCT05155254]). Fixed mRNA vaccines or peptide vaccines offer cost-effectiveness and off-the-shelf availability [65]. In contrast, personalized mRNA vaccines present significant challenges due to their complex manufacturing processes, higher costs, and extended production timelines [65]. Current cancer vaccines are well tolerated, and adverse events predominantly comprise flu-like symptoms [66].

#### 4.1.1 Fixed mRNA Vaccines

BNT111 is an intravenously administered, liposomal mRNA vaccine encoding the melanoma-associated antigens tyrosinase, melanoma-associated antigen A3 (MAGE-A3), New York esophageal squamous cell carcinoma 1 (NY-ESO-1), and transmembrane phosphatase with tensin homology (TPTE) [66]. Tyrosinase represents a melanocytic differentiation antigen, whereas MAGE-A3, NY-ESO-1, and TPTE are germline cancer–testis antigens [67,68]. In the phase I LipoMerit trial (NCT02410733), BNT111 was shown to be safe and clinically active in patients with unresectable CM, even in combination with PD-1 inhibition [66]. The subsequent phase II BNT111-01 trial (NCT04526899) employed a three-arm design comparing cemiplimab monotherapy, BNT111 monotherapy, and BNT111 plus cemiplimab. At a median follow-up of 15.6 months, the study met its primary endpoint, with the combination arm achieving an overall response rate (ORR) of 18.1%, exceeding a prespecified historical benchmark ORR of 10% [69]. However, BNT111 monotherapy also demonstrated clinically meaningful activity with an ORR of 17.4% [69].

#### 4.1.2 Individualized mRNA Vaccines

Exposure to carcinogens or ultraviolet radiation, as well as DNA repair defects, can induce patient-specific mutations that give rise to neoantigens unique to each individual [70]. Based on Moderna’s mRNA platform, the highly individualized cancer vaccine V940 (mRNA-4157), encoding up to 34 neoantigens, was developed as a personalized immunotherapy approach. Neoantigens are tumor-specific antigens arising from genetic alterations that generate peptides not expressed in normal tissues, enabling immune recognition as non-self [71]. Proof of concept was demonstrated in the randomized phase II KEYNOTE-942 trial in patients with high-risk stage IIB or higher cutaneous melanoma [72]. V940 added to adjuvant pembrolizumab improved distant metastasis-free survival and showed a trend toward prolonged progression-free survival without introducing new safety signals, with adverse events mainly consisting of flu-like symptoms [72]. Therefore, the INTerpath-001 trial was set up as larger phase III registrational trial in resected high-risk CM (NCT05933577). First study results are expected to be presented next year. Additionally, the INTerpath-012 trial (NCT06961006) has been started to investigate the efficacy of V940 in advanced, unresectable CM. Last, the INTerpath programme investigating V940 as individualized cancer vaccine has been expanded to multiple tumor entities.

A competing program by BioNTech and Genentech is the development of autogene cevumeran, RO7198457, a personalized mRNA vaccine targeting up to 20 neoantigens. A phase I trial evaluating autogene cevumeran as monotherapy and in combination with the PD-L1-inhibitor atezolizumab demonstrated safety and T cell responses in 71% of patients [73]. Therefore, autogene cevumeran advanced to a phase II trial in unresectable or metastatic CM (NCT03815058). In this trial, cevumeran was tested as mono-therapy or in combination with pembrolizumab. However, the trial’s primary endpoint was not met with a median PFS of 8.3 months in the combination in comparison to 7.9 months with pembrolizumab monotherapy [74]. Subgroup analyses revealed a trend towards improved PFS for CM with low tumor mutational burden or immune responses to multiple neoantigen targets in the combination arm versus pembrolizumab monotherapy [74].

#### 4.1.3 Influences of COVID-19 mRNA Vaccines

Lately, the study of Grippin and colleagues investigated the effect of SARS-CoV-2 mRNA vaccines on the efficacy of ICI [75]. It demonstrated that mRNA vaccines boost efficacy of ICI, even when encoding non-tumor antigens inducing a strong type I interferon–driven reshaping of the tumor microenvironment. In preclinical models and retrospective clinical cohorts, administration of COVID-19 mRNA vaccines within about 100 days of ICI initiation improved both OS and PFS in CM and non-small cell lung cancer. These effects were not seen with conventional vaccines, such as pneumonia or influenza, or in patients treated with chemotherapy. Mechanistically, these vaccines activated innate immunity, primed antigen-presenting cells, expanded tumor-reactive CD8^+^ T cells, and increased intratumoral infiltration and PD-L1 expression in a type I interferon–dependent manner, thereby switching immunologically “cold” tumors into ICI-responsive disease. For melanoma, these findings support mRNA vaccination as a potent immunologic adjuvant that helps sensitizing tumors to ICI, independent of the encoded antigen. They underscore the importance of optimizing timing and sequencing of mRNA vaccines relative to ICIs, bolster the rationale for combining mRNA cancer vaccines with ICI in resistant tumors [76], and suggest that off-the-shelf mRNA vaccines may also provide clinically meaningful immune modulation beyond personalized neoantigen formulations. However, these study results should be interpreted cautiously, given the retrospective, single-center design and the relatively small patient cohorts.

### 4.2 Peptide Vaccines

As an alternative to tumor (neo)antigen-directed cancer vaccines, immune-modulatory vaccines are gaining increasing attention. An advanced program in this area is IO102-IO103, a peptide vaccine targeting indoleamine 2,3-dioxygenase (IDO) and programmed death ligand 1 (PD-L1) [77]. These ligands are expressed in the tumor microenvironment and on T cells and can thereby focus antitumor immunity on IDO- or PD-L1-expressing tumor cells or immunosuppressive cells [78,79]. In preclinical models, addition of an IDO-directed peptide vaccine enhanced PD-1 inhibition, promoting a predominantly T_H1_-driven immune response and remodeling the immunosuppressive tumor microenvironment [80]. In a phase I/II trial in thirty patients with advanced CM (NCT03047928), IO102-IO103 combined with nivolumab achieved an ORR of 80% and a complete response rate of 43%, with vaccine-specific immune responses detectable in both peripheral blood and tumor tissue [77]. Longterm follow-up at approximately 2.5 years showed durable disease control and suggested particular benefit in patients with PD-L1-negative tumors, elevated lactate dehydrogenase (LDH), or liver metastases [81]. More recently, a randomized phase III study in 407 patients (NCT05155254) reported an improvement PFS of 8.4 months, narrowly missing the predefined threshold for statistical significance, while still favoring the combination over pembrolizumab monotherapy in PD-L1–negative or high-LDH subgroups [82]. The IO102-IO103 development program is ongoing and now includes trials assessing the vaccine as neoadjuvant and adjuvant therapy in CM and cutaneous squamous cell carcinoma (NCT05280314), as well as a phase II study (NCT05912244) testing its addition to dual ICI with nivolumab and relatlimab [83].

## 5 Bispecific Antibodies

To address the limitations of conventional ICI, bsAbs were engineered. These molecules simultaneously engage two targets. One example is targeting a tumor antigen with one arm and a T cell protein, typically CD3, with the other, to enhance T cell cytotoxicity toward tumor cells [84]. Another interesting approach is targeting two immune checkpoints in the manner of a dual ICI. In general, this antibody design can be extended to a plethora of extracellular targets. Consequently, immune-desert tumors may be converted into immune-infiltrated lesions and a localized immune activation within the tumor microenvironment may be triggered [85]. However, in solid tumors the limited number of truly tumor-specific antigens remains a major constraint for this approach [84].

Immune-mobilizing monoclonal T cell receptors against cancer (ImmTACs) are a specific type of T cell–engaging bsAbs. These fusion proteins incorporate a high-affinity T cell receptor (TCR) that recognizes peptides presented via HLA complexes of the target cells and couple this recognition to CD3 engagement [86]. The pioneer of this class, tebentafusp, targets the glycoprotein 100 (gp100), presented by HLA-A02:01, with its affinity-enhanced TCR and recruits T cells through its anti-CD3 effector domain [87]. It is approved for treating metastatic uveal melanoma patients, because it has demonstrated a significant enhancement in OS in comparison to the control group with pembrolizumab, ipilimumab, or dacarbazine monotherapy [87]. 3-year follow up data confirm the superiority of tebentafusp with an OS of 27% as related to 18% in the control cohort [88]. Furthermore, the safety profile is manageable dominated by cytokine release syndrome and skin toxicity, typically controllable with step up dosing, fluids, and corticosteroids [88]. Tebentafusp is further investiagted in the second-line treatment of metastatic CM, both as single agent and combined with pembrolizumab, and investigator’s choice, typically ICI, as comparator (NCT05549297).

Brenetafusp (IMC-F106C) is a PRAME-specific ImmTAC developed for HLA-A*02:01-positive patients with advanced CM [89]. Its efficacy and safety were demonstrated in a small phase I study (NCT04262466) with 46 patients with CM who had progressed under ICI [89]. Currently the large phase III PRISM-MEL-301 trial (NCT06112314) is being conducted, which tests two brenetafusp dosing regimens in combination with nivolumab, compared with nivolumab monotherapy or nivolumab plus relatlimab [90]. Furthermore, a competing phase I/II trial is currently ongoing investigating IMA402, a bsAbs against PRAME, in HLA-A*02:01-positive patients with refractory or recurrent solid tumors, including CM (NCT05958121).

Furthermore, multiple early-phase clinical trials are evaluating bsAbs targeting combinations of two immune checkpoints, such as LAG-3 and PD-1 (NCT05577182, NCT04140500), PD-1 and CTLA-4 (NCT03761017, NCT04172454, NCT04606472), PD-1 and TIM-3 (NCT03708328), and PD-L1 and 4-1BB (NCT06984328). In addition, rilvegostomig is a bsAb targeting TIGIT and PD-1 and is currently being investigated in a phase I/II trial in combination with AZD6750, a CD8-guided IL-2 agent (NCT07115043).

Other targets under investigation in early clinical or preclinical trials include Melanoma Antigen Gene (MAGE)-A4 and MAGE-A8. Recently, IMA401, a bsAb targeting the HLA-A*02:01-presented MAGE-A4/8 antigen(s), has entered a phase I basket trial in solid tumors (NCT05359445). Moreover, BNT326, an antibody-drug conjugate targeting HER-2, is evaluated as single agent and combined with BNT327, a bsAb against PD-L1 and VEGF-A (NCT07070232) in a phase I/II basket trial that includes CM. Furthermore, GI-102 (NCT05824975), a bsAb against CD80 and IL2Rβγ, NVG-111 (NCT04763083), a bsAB against ROR1 and CD3, and JMT108 (NCT07317505), a bsAb against PD-1 and IL 15, are analyzed in early phase clinical trials. Last, a phase I first-in-human study is assessing the tolerability and safety of FS222, a bsAb against CD137 and PD-L1 (NCT04740424).

Despite this promise, a key limitation of bsAbs must be discussed: most of them are restricted to the HLA-A*02:01 genotype, which is present in only about half of Caucasians, thereby excluding a significant number of patients from treatment [91]. Moreover, targets must be expressed on the extracellular surface of the target cells to enable binding of bsAbs. Furthermore, immunosuppressive microenvironments may decrease treatment responses.

## 6 Cellular Therapies

### 6.1 Tumor Infiltrating Leukocytes (TILs)

Even in later therapy line, adoptive cell therapy (ACT) with tumor-infiltrating leukocytes (TILs) is an appealing treatment option for patients with advanced CM [92]. Early evidence has been provided by Steve Rosenberg and his group in 1994 [93]. Today’s concept has been developed and fine-tuned by leading medical centers, such as the National Cancer Institute in the USA, the University of Copenhagen in Denmark or the Sheba Medical Center in Israel [94,95,96]. In principle, TILs are isolated from patient tumor samples, expanded *ex vivo* at specialized facilities, and a frozen individualized TIL product is returned to the hospitals [97]. Subsequent to a lymphodepleting chemotherapy patients receive TILs, followed by several Interleukin-2 injections and sometimes in combination with PD-1-inhibition [97]. Early clinical phase I to II trials demonstrated ORR from 28 to 58% [98,99,100,101,102]. In 2024, lifileucel was approved by the U.S. Food and Drug Administration as the first TIL-ACT for advanced CM previously treated with ICI or, in the case of *BRAF*-mutant disease, TT. An ORR of 31.4% was reported in a large multicenter phase II study including 153 patients treated with lifileucel, which was administered on average as a fourth-line therapy [103]. The 5-year analysis of the C-144-01 study of lifileucel (NCT02360579) reported an OS of 19.7% and a median duration of response of 36.5 months, and an ongoing response in around one third of all responders at the time of analysis [104]. Furthermore, a multicenter, phase III study of patients with CM at the Netherlands Cancer Institute (NCT02278887) who were refractory to PD-1 inhibition demonstrated that PFS improved significantly from 3.1 months in the ipilimumab cohort to 7.2 months in the TIL group [105]. An ORR of 49% was reported for the TIL cohort, whereas 21% of patients responded to ipilimumab [105]. However, responses to TIL-ACT come at a cost, since grade ≥ 3 adverse events, typically cytopenias, fever, chills, dyspnea, are frequent because of the lymphodepleting chemotherapy and Interleukin-2 infusions [104,105]. As the response to TIL monotherapy is strongly reduced by pre-treatment with PD-1 inihibition or TT, it is also debated whether it should be assessed as first-line treatment [106]. Therefore, several phase I and II trials investigate the application of TILs in combination with PD-1 inhibition to enhance therapy efficacy (e.g., NCT03638375, NCT03475134, NCT04165967). The largest TIL trial is currently underway. This phase III study involves 670 patients and compares the combination of lifileucel and pembrolizumab with pembrolizumab alone in treatment-naive patients with advanced CM (NCT05727904).

### 6.2 Engineered T Cells

In advanced-stage hematological malignancies, ACT with genetically engineered T cells showed promising efficacy [107,108]. Nevertheless, translation to solid tumors, including CM, remains at early clinical, experimental stages and is a major challenge [109]. Especially, the tumor microenvironment in solid tumors creates multiple hurdles for T cells, since it serves as a physical barrier to infiltration and fosters an immunosuppressive environment that diminishes their anti-tumor effects [109]. The tumoral stroma is composed of cancer-associated fibroblasts, which also foster an immunosuppressive milieu [110]. Moreover, other immune cells such as MDSCs, regulatory T cells, neutrophils, or macrophages build a regulatory network that may be tumor-promoting [28,111]. Lastly, this immunosuppressive microenvironment directly influences the fitness of engineered T cells by promoting T cell exhaustion, which must be accounted for when designing T cells [112]. Engineered T cells can be classified as T cells with genetically modified T cell receptors (TCR-T cells) and chimeric antigen receptor (CAR) T cells [113]. These two types differ in how they recognize antigens. TCR-T cells detect peptide antigens that are presented via major histocompatibility complexes (MHC), while CAR-T cells recognize surface targets independently of MHC [113].

Several smaller preclinical trials are studying TCR-T cells to enhance recognition of melanoma antigens, including NY-ESO-1 (NCT06942143, NCT06889766, NCT05296564, and NCT02650986) and MAGE-C2 (NCT04729543). In a phase I/II trial of IMA203 (NCT03686124), which targets PRAME with TCR-T cells, the ORR was 52.5% in 40 patients with advanced solid tumors, including CM [114]. The confirmed ORR was 28.9%, with a median response duration of 4.4 months [114]. Therefore, IMA203 is being studied in a larger phase III clinical trial with 360 patients with refractory advanced CM and compared to investigator’s choice (NCT06743126) [115]. Besides, a preclinical phase I study is analyzing whether the response to IMA203 may be enhanced by mRNA-4203, an investigational mRNA to enhance to antigen response to PRAME [116].

A small phase I interventional study investigating CAR-T cells reactive to interleukin 13 receptor subunit alpha 2 (IL13Ralpha2) has recently started (NCT04119024). Furthermore, a preclinical study is analyzing the safety and efficacy of CAR T-cells targeting CD19 or CD20 in multiple cancer entities, including CM (NCT06508775). An approach targeting vascular endothelial growth factor receptor 2 (VEGFR2) by CAR T-cells has not been successful, as most patients progressed (NCT01218867). Major challenges in CAR T-cell therapy remain such as targeting the right tumor antigen, off-tumor side effects, heterogeneity of tumors, antigen loss of tumor cells or the immunosuppressive tumor microenvironment [117].

## 7 Intralesional Therapies

The concept of intralesional therapy aims to enhance both local and systemic anti-tumor responses by *in situ* application of immunostimulatory agents such as viruses or cytokines. T-VEC has been the first oncolytic virus approved for treating unresectable CM [118]. Neoadjuvant application of T-VEC (NCT02211131) significantly improves PFS and OS in melanoma patients with slowly growing cutaneous or nodal lesions as compared to surgery alone, as demonstrated in a phase II trial [119,120]. Since non-injected lesions exhibit increased intratumoral CD8^+^ T cells during T-VEC application, a combination of T-VEC with a dual ICI with nivolumab and ipilimumab has been investigated [121,122]. However, adding T-VEC to nivolumab and ipilimumab does not enhance PFS or OS in comparison to dual ICI alone [122].

Daromun is a combination of two immunocytokines, L19IL2 and L19IFN [123]. These cytokines are fused to the monoclonal antibody L19 which detects an extracellular domain of fibronectin. In a phase II study, Daromun demonstrated disease control in cutaneous or nodal lesions of CM [123]. Therefore, it was transferred into a phase III trial investigating the neoadjuvant application of Daromun in patients with stage IIIB/C CM (NCT02938299) [124]. The neoadjuvant intralesional application of Daromun and the subsequent resection of the lesions leads to a significant enhancement in RFS and DMFS compared to surgery alone and shows an acceptable safety profile [124]. Regulatory decisions from major agencies, including the FDA and EMA, are currently awaited.

Vusolimogene oderparepvec (RP1), another genetically engineered herpes virus type 1, was investigated in the phase I/II IGNYTE study as second-line intralesional therapy in combination with nivolumab after progression under PD-1-inhibition (NCT03767348) [125]. The combination of RP1 with nivolumab achieved an ORR of 32.9%. This included patients with negative prognostic factors such as PD-L1 negativity [125]. Moreover, a phase I trial investigates whether RP1 injected into the primary tumor site around one month prior to sentinel lymph node biopsy may reduce sentinel lymph node positivity (NCT06216938). Last, a large, randomized phase III trial comparing RP1 in combination with nivolumab against investigator’s choice, consisting of ICI and chemotherapy, has started for patients refractory to ICI (NCT06264180).

Other oncolytic viruses, including OH2, an oncolytic herpes virus type II, are currently undergoing early phase clinical trials [126]. Oncolytic viral therapies may pose an interesting treatment approach for selected patient populations.

## 8 Future Directions

Current NCCN and ESMO guidelines for CM treatment recommend initiating ICI as first-line therapy in advanced CM, regardless of *BRAF* mutation status [50,127]. This is based on the randomized controlled trials SECOMBIT and DREAMseq on the treatment sequencing of ICI and TT [128,129]. Although neither trial was powered to demonstrate significant differences between the arms, both trials indicate superiority of first-line ICI followed by second-line TT over first-line TT and second-line ICI. However, *BRAF*-mutant patients with high tumor burden may be offered first-line TT for tumor debulking [50,127]. Regarding adjuvant treatment decisions in stage III CM, there is debate over whether adjuvant TT should be preferred over adjuvant ICI for *BRAF*-mutant patients, as several studies have demonstrated the superiority of TT [14,15,16]. In our opinion, this should be openly discussed during shared decision-making regarding adjuvant therapy with *BRAF*-mutant melanoma patients in stage III. Combinatorial approaches are also appealing to prevent resistance to TT or ICI. Targets that have been addressed in experimental studies include focal adhesion kinase, epidermal growth factor receptor, or cyclin-dependent kinase 4/6 (CDK4/6) inhibitors [130,131,132]. Nevertheless, large randomized controlled trials are needed.

Treatment personalization will be crucial to build on the success in CM treatment in this decade and to navigate through the numerous treatment options to date. To this end, reliable predictive biomarkers will have a central role. These can be classified into tissue-derived factors, circulating blood-based markers, or other factors. An important tissue-based biomarker of clinical response is the percentage of TILs in CM [133]. Besides, the expression of immune checkpoints or their respective ligands has been extensively analyzed. PD-L1 overexpression was the first biomarker to correlate with treatment response [26,134]. However, this correlation depends strongly on the tissue analyzed, as PD-L1 expression in lymph nodes is more strongly associated with treatment responses than expression in CM or metastases [135]. Altogether, PD-L1 is a controversially discussed, as treatment responses are observed in PD-L1 low CM [136]. In contrast, expression of either LAG-3 or TIGIT on tumor-infiltrating leukocytes strongly correlates with poor survival, whereas PD-1 expression does not [137]. Next, there is evidence that certain gene expression profiles correlate with improved OS. High Interferon-γ signatures correlate with improved therapy responses in neoadjuvant, adjuvant and palliative therapies [138,139,140,141]. Additionally, a high tumor mutational burden promotes an enhanced response to ICI [27,142]. Meanwhile, several diagnostic tests for gene-expression profiling in CM are available, such as the nine-gene signature MelaGenix score and the eight-gene signature Merlin score [143,144]. The MelaGenix score is currently being investigated in the NivoMela trial for stage IIA to IIC CM (NCT04309409) to select high-risk patients for an adjuvant therapy with Nivolumab. Recently, the MERLIN_001 trial demonstrated that CM, tumor stage T1 to T3, with a low risk of sentinel lymph node metastasis can be reliably identified with the Merlin gene expression profile [144]. Therefore, the authors suggest that this should be implemented in shared decision-making regarding sentinel lymph node biopsy [144].

Among currently applied blood-based biomarkers, circulating tumor DNA (ctDNA) stands out its high sensitivity and predictive value. Therefore, ctDNA may be used not only for the surveillance of CM patients but also for the prediction of relapses during neoadjuvant, adjuvant, or palliative treatment [72,88,145,146,147]. Other blood biomarkers include immune cell populations, such as neutrophils, monocytes or eosinophils, or cytokine levels, such as Interleukin-6 or Interleukin-8, among many other factors [148,149,150,151,152]. While high neutrophil counts are associated with poor prognosis and disease progression, eosinophils have a protective role and are considered a beneficial prognostic factor [149,150,151,152]. Moreover, in non-responders to ICI higher baseline levels of Interleukin-6, Interleukin-8, and MDSC counts were found [148].

The gut microbiome attracted considerable attention in oncoimmunology, as therapy responses have been attributed to the presence of certain bacterial strains, such as Akkermansia muciniphila, and have been positively influenced by fecal microbiota transplantation (FMT) [153,154,155,156]. Meanwhile, phase I and II studies demonstrated safety and clinical activity of FMT for cancer patients [157,158]. Moreover, high dietary fiber intake has a beneficial effect on the gut microbiome, reflecting improved therapy outcome [159]. Therefore, the gut microbiome is currently targeted in numerous clinical studies not only to further improve therapy outcomes in cancer patients (e.g., NCT05251389, NCT06623461, NCT05286294), but also to treat immune mediated side effects (e.g., NCT04038619, NCT03819296). Additionally, this will be a focus of diet intervention studies [160].

Last, there are several other prognostic factors that influence CM survival or treatment response, for example body composition or gender [161,162,163].

Altogether, predictive biomarkers have great potential. But there is also strong need for biomarker-driven clinical trials to both validate these in prospective studies and investigate how they will facilitate treatment decisions.

## 9 Conclusion

Over the past decade, the treatment of advanced CM was revolutionized by both ICI and TT [25,164]. Nevertheless, substantial challenges persist, including immune-resistant tumor microenvironments in brain and liver metastases [34,35,36,37], therapy-refractory CM [21,22] and the management of locoregional and subcutaneous metastases [165]. Emerging strategies, such as next-generation checkpoint inhibitors, cancer vaccines, bispecific antibodies, and cellular therapies, as well as neoadjuvant treatment concepts, show promise in further improving outcomes by deepening and prolonging responses, overcoming resistance, and preventing metastasis. In addition, several other promising targets are currently being investigated in early-phase experimental studies (Supplementary Table S3). However, future work will require rational combination strategies, biomarker-driven patient selection, and carefully designed clinical trials to translate these innovative approaches into long-lasting benefits for patients with melanoma (Fig. 2).

## Data Availability

Not applicable.

## Supplementary Materials

The supplementary material is available online at https://www.techscience.com/doi/10.32604/or.2026.078650/s1.

## Abbreviations

ACT	Adoptive cell therapy
bsAb	Bispecific antibody
BTLA	B and T lymphocyte attenuator
CAR	Chimeric antigen receptor
CDK4/6	Cyclin-dependent kinase 4/6
CM	Cutaneous melanoma
CTLA-4	Cytotoxic T-lymphocyte-associated protein 4
DCR	Disease control rate
DMFS	Distant metastasis-free survival
EGFR	Epidermal growth factor receptor
EMA	European Medicines Agency
ESMO	European Society For Medical Oncology
EFS	Event-free survival
FDA	Food and Drug Administration
FMT	Fecal microbiota transplantation
gp100	Glycoprotein 100
ICI	Immune checkpoint inhibitor
IDO	Indoleamine 2,3-dioxygenase
ImmTAC	Immune-mobilizing monoclonal T cell receptor against cancer
ITT	Intention-to-treat
LAG-3	Lymphocyte activation gene 3
LDH	Lactate dehydrogenase
MAGE-A3	Melanoma-associated antigen A3
MAGE-A4	Melanoma-associated antigen A4
MAGE-A8	Melanoma-associated antigen A8
MAGE-C2	Melanoma-associated antigen C2
MAPK	Mitogen-activated protein kinase
MDSCs	Myeloid-derived suppressor cells
MHC	Major histocompatibility complex
NCCN	National Comprehensive Cancer Network
NY-ESO-1	New York esophageal squamous cell carcinoma 1
ORR	Overall response rate
OS	Overall survival
pCR	Pathological complete response
PD-1	Programmed cell death protein 1
PD-L1	Programmed death ligand 1
PFS	Progression-free survival
PRAME	Preferentially expressed antigen in melanoma
RFS	Relapse-free survival
RP1	Vusolimogene oderparepvec
TCR	T cell receptor
TIL	Tumor-infiltrating lymphocyte
TIM-3	T cell immunoglobulin and mucin-domain containing-3
TIGIT	T cell immunoglobulin and ITIM domain
TT	Targeted therapy
TPTE	Transmembrane phosphatase with tensin homology
T-VEC	Talimogene laherparepvec
VEGF-A	Vascular endothelial growth factor A
VEGFR2	Vascular endothelial growth factor receptor 2
VISTA	V-domain Ig suppressor of T cell activation
