# Supplementary material for: After a Decade of Therapy Revolution in Cutaneous Melanoma—Perspectives on Emerging Treatment Strategies
Source: Oncol Res. 2026 May 21;34(6):3. doi: 10.32604/or.2026.078650 (PMC13223291; doi:10.32604/or.2026.078650)
Supplement: Supplementary file 1 [file OncolRes-34-78650-s001.zip › TSP_OR_78650-s001.docx]

Supplementary Tables

**Table S1:** A selection of completed phase III clinical trials in cutaneous melanoma (CM)

| **NCT number** | **Therapeutic target** | **Study arms and sample sizes** | **Trial phase** | **Study timeline** | **Preliminary efficacy data** |
| --- | --- | --- | --- | --- | --- |
| **Unresectable CM** | | | | | |
| NCT01844505 (CheckMate 067) | PD-1 and CTLA-4 (dual ICI) | Nivolumab + Ipilimumab (314) vs. Nivolumab (316) vs. Ipilimumab (315) | 3 | 2013 - 2024 | 10-yr ^1^: ORR: 58.3% (N+I), 44.9% (N), 19.0% (I)  OS: 43% (N+I), 37% (N), and 19% (I) |
| NCT01909453 (COLUMBUS) | BRAF and MEK inhibition (TT) | Encorafenib + Binimetinib (192) vs. Vemurafenib (191) vs. Encorafenib (194) | 3 | 2013-2024 | 7-yr ^2^:  PFS: 21.2% (E+B), 6.4% (V), 15.8 % (B)  OS: 27.4% (E+B), 18.2% (V), 31.7% (E)  ORR: 64.1% (E+B), 40.8% (V), 51.5% (B) |
| NCT01584648 (**COMBI-d**),  NCT01597908 (**COMBI-v)** | BRAF and MEK inhibition (TT) | **COMBI-d:** Dabrafenib + Trametinib (211) vs. Dabrafenib + Placebo (212) | 3 | **COMBI-d:**  2012 - 2019 | 5-yr ^3^:  **COMBI-d:**  PFS: 17% (D+T) vs. 13% (D+P)  OS: 32% (D+T) vs. 27% (D+P) |
|  |  | **COMBI-v:** Dabrafenib + Trametinib (352) vs. Vemurafenib (352) |  | **COMBI-v:**  2012 - 2019 | **COMBI-v:**  PFS: 20% (D+T) vs. 9% (D+P)  OS: 36% (D+T) vs. 23% (D+P) |
| NCT03470922 (RELATIVITY-047) | PD-1 and LAG-3 (dual ICI) | Nivolumab + Relatlimab (355) vs. Nivolumab mono (359) | 3 | 2018 – 2030 (est.) | 3-yr ^4^: PFS: 10.2 months (N+R), 4.6 months (N)  OS: 54.6% (N+R), 48.0% (N)  ORR: 43.7% (N+R), 33.7% (N) |
| **Resectable CM (adjuvant therapy)** | | | | | |
| NCT01682083 (COMBI-AD) | BRAF and MEK inhibition (TT) | Dabrafenib + Trametinib (435) vs. Placebo (432); CM stage ≥ IIIA | 3 | 2013 - 2023 | 8-yr ^5^:  OS: 71% (D+T), 65% (P)  RFS: 44% (D+T), 61% (P)  DMFS: 28% (D+T), 37% (P) |
| NCT02388906 (CheckMate 238) | PD-1 (ICI) | Nivolumab (453) vs. Ipilimumab (453);  CM stage ≥ IIIB | 3 | 2015 - 2024 | 5-yr ^6^:  RFS: 50% (N), 39% (I) DMFS: 58% (N), 51% (I) |
| NCT02362594 (KEYNOTE-054) | PD-1 (ICI) | Pembrolizumab (514) vs. Placebo (505); CM stage ≥ IIIA | 3 | 2015 - 2026 | 7-yr ^7^:  RFS: 50% (Pembro), 36% (Placebo) DMFS: 54% (Pembro), 42% (Placebo) |
| NCT03553836 (KEYNOTE-716) | PD-1 (ICI) | Pembrolizumab (487) vs. Placebo (489); CM stage ≥ IIB | 3 | 2018 - 2033 | 3-yr (estimated) ^8^:  RFS: 76.2% (Pembro), 63.4% (Placebo)  DMFS: 84.4% (Pembro), 74.7% (Placebo) |
| NCT04099251 (CheckMate 76K) | PD-1 (ICI) | Nivolumab (526) vs. Placebo (264) | 3 | 2019 - 2027 | 1-yr ^9^:  RFS: 89.0% (N), 79.4% (P)  DMFS: 92.3% (N), 86.7% (P) |
| NCT05002569 (RELATIVITY-098) | PD-1 + LAG-3 (dual ICI) | Nivolumab + Relatlimab (547) vs. Nivolumab (546); CM stage ≥ IIIA | 3 | 2021 – 2025  (early termination) | 2-yr ^4^: PFS: 62% (N+R), 64% DMFS: 73% (N+R), 76% (N) |
| **Neoadjuvant treatment** | | | | | |
| NCT04949113 (NADINA) | PD-1 and CTLA-4 (dual ICI) | 2 cycles of neoadjuvant Nivolumab and Ipilimumab followed by adjuvant Nivolumab (212) vs. adjuvant Nivolumab (211); CM stage ≥ IIIB | 3 | 2021 - 2028 (est.) | 1-yr ^10^:  EFS: 83.7% (neoadj.), 57.2% (adj.)  pCR: 47.2% (neoadj.) |

Trial data according to https://www.clinicaltrials.gov, data research from 11 FEB 2026. **Abb:** est. = estimated; PD-1 = Programmed cell death protein 1; CTLA‑4 = Cytotoxic T-lymphocyte-associated protein 4; ICI = Immune checkpoint inhibitor; BRAF = B-Raf proto-oncogene; MEK = Mitogen-activated protein extracellular signal-regulated kinase; TT = targeted therapy; ORR = overall response rate; OS = overall survival; PFS = Progression-Free Survival; RFS = Relapse-Free Survival; EFS = Event-Free Survival; DMFS = Distant Metastasis-Free Survival; pCR = pathological complete response; LAG-3 = Lymphocyte-activation gene 3

**Table S2:** Summary of selected studies on emerging therapies for CM

| **NCT number** | **Therapeutic target & mechanism** | **Drug investigated** | **Study arms and sample sizes** | **Trial phase** | **Preliminary efficacy data** |
| --- | --- | --- | --- | --- | --- |
| **Neoadjuvant treatment** | | | | | |
| NCT03698019 (SWOG S1801) | PD-1 (ICI) | Pembrolizumab | Neoadjuvant tx. (154) vs. adjuvant tx. (159); CM stage ≥ IIIB | 2 | 2-yr ^11^:  EFS: 72% (neoadj.), 49% (adj.)  pCR: 21% (neoadj.) |
| NCT06190951 | LAG-3 and PD-1 (dual ICI) | Cemiplimab (anti-PD-1) and Fianlimab (anti-LAG-3) | Cemilimab + Fianlimab vs. Cemiplimab and Placebo | 2 | N.A. |
| NCT05116202 (Morpheus) | Tobemstomig (bsAb against PD-1 and LAG-3) | Tobemstomig | Neoadjuvant: Tobemstomig (40) vs. Tobemstomig and Tiragolumab (anti-TIGIT) (20) vs. Atezolizumab (anti-PD-L1) + Tiragolumab (20) vs.Nivolumab and Ipilimumab ( 22) CM stage III | 1b/ 2 | pCR: 80% (Tobemstomig), 77.3% (Nivolumab and Ipilimumab) ^12^ |
| **Novel ICIs** | | | | | |
| NCT05352672 | LAG-3 and PD-1 (dual ICI) | Cemiplimab (anti-PD-1) and Fianlimab (anti-LAG-3) | Cemiplimab + Fianlimab high dose vs. Cemiplimab + Fianlimab low dose vs. Pembrolizumab vs. Cemiplimab;  Unresectable CM | 3 | N.A. |
| NCT05608291 | LAG-3 and PD-1 (dual ICI) | Cemiplimab (anti-PD-1) and Fianlimab (anti-LAG-3) | Cemiplimab + Fianlimab high dose vs. Cemiplimab + Fianlimab low dose vs. Pembrolizumab | 3 | N.A. |
| NCT05060432 | TIGIT (ICI) | EOS-448 (anti-TIGIT) | EOS-448 + pembrolizumab or inupadenant (adenosine A2A receptor antagonist) or dostarlimab (anti-PD-1) or inupadenant and dostarlimab or dostarlimab and standard of care chemotherapies vs. inupadenant combined with dostarlimab | 1-2 | N.A. |
| NCT05483400 | TIGIT and PD-L1 (dual ICI) | Tiragolumab | Tiragolumab + atezolizumab (anti-PD-L1) (multi basket trial) | 2 | N.A. |
| NCT05130177 | TIGIT and PD-1 (dual ICI) | Domvanalimab | Domvanalimab + Zimberelimab (anti-PD-1) | 2 | N.A. |
| NCT02608268 | TIM-3 and PD-1 (dual ICI) | Sabatolimab | Sabatolimab (various doses) vs. Sabatolimab (various doses) + Spartalizumab vs. Sabatolimab + chemotherapy | 1-2 | Stable disease: 19% (CM)  Median PFS: 1.8 months for CM ^13^ |
| NCT02817633 (AMBER) | TIM-3 and PD-1 and LAG-3 (dual/ triple ICI) | TSR-022 | TSR-022 vs. TSR-022 + Nivolumab or Dostarlimab or Dostarlimab and Encelimab (anti-LAG-3) or Dostarlimab and chemotherapy | 1 | ORR: 42.9% (PD-1–naïve CM) or 4.7% (relapsed or refractory CM) ^14^ |
| **Cancer vaccines** | | | | | |
| NCT04526899 (BNT111‑01) | BNT111 and PD-1 | BNT111 (fixed mRNA vaccine) | BNT111 + Cemiplimab vs. BNT111 vs. Cemiplimab | 2 | ORR ^15^: 18.1% (BNT111 + Cemiplimab), 17.4% (BNT111 monotherapy), 13.6% (Cemiplimab monotherapy) |
| NCT05933577 (INTerpath-001) | V-940 and PD-1 | V-940 (individualized mRNA vaccine) | V940 + Pembrolizumab vs. Pembrolizumab + Placebo; CM stage ≥ IIB | 3 | N.A. |
| NCT06961006 (INTerpath-012) | V-940 and PD-1 | V-940 (individualized mRNA vaccine) | V940 + Pembrolizumab vs. Pembrolizumab + Placebo; unresectable CM stage ≥ III | 2 | N.A. |
| NCT03815058 | Autogene Cevumeran and PD-1 | Autogene Cevumeran (individualized mRNA vaccine) | Autogene Cevumeran + Pembrolizumab vs. Pembrolizumab; unresectable CM stage ≥ III | 2 | 2-yr ^16^:  Median PFS: 8.3 months (combination), 7.9 months (Pembrolizumab)  OS: 74.17% (combination), 63.88% (Pembrolizumab)  ORR: 41.7% (combination), 48.8% (Pembrolizumab) |
| NCT05155254 | IO102-IO103 and PD-1 | IO102-IO103 (peptide vaccine against IDO and PD‑L1) | IO102-IO103 + Pembrolizumab vs. Pembrolizumab | 3 | Median PFS: 19.4 months (IO102-IO103 + Pembrolizumab), 11.0 months (Pembrolizumab) ^17^ |
| NCT05280314 | IO102-IO103 and PD-1 | IO102-IO103 | Neoadjuvant vs. post-surgery treatment | 2 | N.A. |
| NCT05912244 | IO102-IO103 and PD-1 and LAG-3 | IO102-IO103 | IO102-IO103 + Nivolumab + Relatlimab | 2 | N.A. |
| **BsAbs** | | | | | |
| NCT05549297 (TEBE-AM) | Tebentafusp (gp100-specific ImmTAC) and PD-1 | Tebentafusp | Tebentafusp vs.  Tebentafusp with Pembrolizumab  vs. Investigator’s choice | 3 | N.A. |
| NCT06112314 (PRISM-MEL-301) | Brenetafusp (PRAME-specific ImmTAC) and PD-1 | Brenetafusp | Brenetafusp low dose + Nivolumab vs. Brenetafusp high dose + Nivolumab vs. Nivolumab or Nivolumab and Relatlimab | 3 | N.A. |
| NCT05958121 | IMA402 (PRAME-specific bsAb) | IMA402 | IMA402 (dose escalation and extension) | 1-2 | N.A. |
| NCT05577182 | INCA32459 (LAG-3 and PD-1 bsAb) | INCA32459 | INCA32459 (tolerability and dose finding) | 1 | N.A. |
| NCT04140500 | RO7247669 (LAG-3 and PD-1 bsAb) | RO7247669 | RO7247669 (dose escalation and expansion cohorts) | 1-2 | ORR: 17.1% Disease control rate (DCR): 51.4% ^18^ |
| NCT06984328 | Acasunlimab (bsAb against PD-L1 and 4‑1BB) | Acasunlimab | Acasunlimab and Pembrolizumab vs. Pembrolizumab | 2 | N.A. |
| NCT03761017 | Lorigerlimab (bsAb against PD-1 and CTLA-4) | Lorigerlimab | Lorigerlimab (tolerability and dose finding) | 1 | N.A. for CM |
| NCT04172454 | AK104 (bsAb against PD-1 and CTLA-4) | AK104 | AK104 (tolerability and dose finding) | 1-2 | N.A. for CM |
| NCT04606472 | SI-B003 (bsAb against PD-1 and CTLA-4) | SI-B003 | SI-B003 (tolerability and dose finding) (60) | 1 | ORR: 16.1% DCR: 50.0% ^19^ |
| NCT03708328 | Lomvastomig (bsAb against PD-1 and TIM-3) | Lomvastomig | Lomvastomig (dose escalation and expansion) | 1 | N.A. |
| NCT05824975 | GI-102 (bsAb against CD80 and IL2Rβγ) | GI-102 | GI-102 monotherapy vs. combination with conventional anti-cancer drugs, Pembrolizumab or Trastuzumab Deruxtecan | 1-2 | N.A. |
| NCT04763083 | NVG-111 (bsAB against ROR1 and CD3) | NVG-111 | NVG-111 (hematological malignancies and solid tumors) | 1 | N.A. for CM |
| NCT07317505 | JMT108 (bsAb against PD-1 and IL‑15) | JMT108 | JMT108 (dose escalation and expansion) | 1 | N.A. |
| NCT05359445 | IMA401 (MAGE-A4/ 8-specific bsAb) | IMA401 | IMA402 or IMA402 + Pembrolizumab | 1 | N.A. |
| NCT07070232 | BNT326 (antibody drug conjugate targeting HER3) and BNT327 (bsAb against PD-L1 and VEGF-A) | BNT326, BNT327 | Basket trial with multiple cancer entities, dosing and combinations of BNT326 and BNT327 will be investigated | 1-2 | N.A. |
| NCT07115043 | TIGIT + PD-1 (ICI) +/- IL-2 | Rilvegostomig (PD1-TIGIT bsAb), AZD6750 (CD8 guided IL-2) | AZD6750 + Rilvegostomig vs. AZD6750 | 1-2 | N.A. |
| NCT04740424 | FS222 (bsAB against CD137/ PD‑L1) | FS222 | Dose finding | 1 | N.A. |
| **Cellular therapies** | | | | | |
| NCT02360579 (C-144-01) | Lifileucel (TIL-ACT) | Lifileucel | Second-line treatment for advanced CM | 2 | 5-yr ^20^: OS: 19.7 % Median duration of response: 36.5 months |
| NCT05727904 | Lifileucel (TIL-ACT) and PD-1 | Lifileucel | Lifileucel and Pembrolizumab vs. Pembrolizumab (option for crossover); unresectable CM stage ≥ IIIC, first-line therapy | 3 | N.A. |
| NCT02278887 | TILs | TILs | TILs vs. Ipilimumab | 3 | PFS: 7.2 months (TIL), 3.1 months (Ipilimumab) Objective response rate: 49% (TIL), 21% (Ipilimumab) Median overall survival (OS): 25.8 months (TIL), 18.9 months (Ipilimumab) ^21^ |
| NCT06743126 | IMA203 (Anzucel) (PRAME-directed TCR-T cells) | IMA203 | IMA203 vs. investigator’s choice | 3 | N.A. |
| NCT06942143 | Super1 TCR-T (TCR T cells) | NY-ESO | ACT with Super1 TCR-T | 1 | N.A. |
| NCT06889766 | NY-ESO1 TCR redirected autologous T cell product | NY-ESO | ACT with LauT-1 | 1 | N.A. |
| NCT05296564 | HBI 0201-ESO TCRT (Anti-NY-ESO-1 TCR-transduced peripheral blood lymphocytes) | NY-ESO | ACT with HBI 0201-ESO | 1-2 | N.A. |
| NCT02650986 | autologous NY-ESO-1 TCR/dnTGFbetaRII transgenic T cells | NY-ESO | ACT with TGFbDNRII-transduced autologous tumor infiltrating lymphocytes | 1-2 | N.A. |
| NCT04729543 | MAGE-C2/HLA-A2 TCR T cells (MC2 TCR T cells) | MAGE-C2 | ACT with MC2 TCR T cells | 1-2 | N.A. |
| NCT04119024 | IL13Ralpha2 CAR T cells | IL13Ralpha2 | ACT with IL13Ralpha2 CAR T cells | 1 | N.A. |
| NCT06508775 | MB-CART19.1 (CAR T cells against CD19) | CD19 or CD20 | ACT with MB-CART19.1 or MB-CART20.1 or MB-CART2019.1 | N.A. | N.A. |
|  | MB-CART20.1 (CAR T cells against CD20) |  |  |  |  |
|  | MB-CART2019.1 (CAR T cells against CD19 and CD20) |  |  |  |  |
| **Intralesional therapies** | | | | | |
| NCT02938299 (PIVOTAL) | Daromun (combination of two immunocytokines, L19IL2 and L19IFN) | Daromun | Daromun neoadjuvant (for four weeks) + surgery vs. surgery alone | 3 | 2-yr ^22^:  RFS: 41.6% (Daromun + surgery) vs. 23.6% (surgery)  DMFS: 59.4% (Daromun + surgery) vs. 38.8% (surgery) |
| NCT03767348 (IGNYTE) | Vusolimogene Oderparepvec (genetically engineered herpes virus type 1) | Vusolimogene Oderparepvec | Vusolimogene Oderparepvec vs. Vusolimogene Oderparepvec and Nivolumab (multiple tumor entities) | 2 | ORR: 33.6%  Median PFS: 3.6 months (all patients), 35.5 months (responders), 1.9 months (nonresponders)  Median OS: not reached ^23^ |
| NCT06264180 (IGNYTE-3) | Vusolimogene Oderparepvec and Nivolumab | Vusolimogene Oderparepvec | Vusolimogene Oderparepvec and Nivolumab vs. investigator’s choice (ICI or chemotherapy), second-line therapy in advanced CM | 3 | N.A. |

Trial data according to https://www.clinicaltrials.gov, data research from 12 FEB 2026. **Abb:** LAG-3 = Lymphocyte-activation gene 3; TIGIT = T cell immunoreceptor with Ig and ITIM domains; TIM-3 = T-cell immunoglobulin and mucin-domain containing-3; CD = Cluster of Differentiation; ROR1 = Receptor Tyrosine Kinase-like Orphan Receptor 1; IL = Interleukin; MAGE = Melanoma-Associated Antigen Gene; HER3 = Human Epidermal Growth Factor Receptor 3; VEGF = Vascular Endothelial Growth Factor; TIL-ACT = adoptive cell transfer with tumor infiltrating leukocytes; TCR = T-cell receptor; ACT = Adoptive Cell Transfer; N.A. = not available; ORR = overall response rate; ICI = Immune checkpoint inhibitor; OS = overall survival; PFS = Progression-Free Survival; pCR = pathological complete response; EFS = Event-Free Survival; PD-1 = Programmed cell death protein 1

**Table S3:** Examples of other interesting early-phase therapeutic agents in melanoma treatment. Drugs were administered alone or in combination with others, such as ICI.

| **Drug** | **Details** |
| --- | --- |
| DNA vaccine platforms | TRP-2 and gp100-directed DNA vaccines SCIB1 / iSCIB1+ generate strong T cell responses against melanoma (NCT04079166) ^24^. |
| mRNAs encoding for cytokines | SAR441000 (mixture of four mRNAs encoding single-chain IFN‑α‑2b, IL-12, IL-15 sushi domain, and GM-CSF) was successfully applied in some melanoma patients (NCT03871348) ^25^. |
| CDK4/6 inhibitors | The CDK4/6 pathway is crucial for cell cycle regulation. As it is frequently dysregulated in melanoma, it presents an appealing target for therapeutic intervention ^26^. Besides, it might be an interesting addition to ICI to overcome therapy resistances ^27^. |
| pan-RAF inhibitors | The application of different pan-RAF inhibitors is currently under clinical investigation in *NRAS*-mutant CM ^28^. |
| Cytokine-based therapies | Nemvaleukin alfa (ALKS 4230, selective IL-2 pathway activator) or IL-15 superagonists are currently under investigation (NCT02799095)^29^. |
| Epigenetic & transcription-targeting drugs | HDAC inhibitors and DNA methylation modulators are currently investigated in clinical trials (e.g., NCT02697630, NCT0467468) ^30, 31^. |

**Abb:** IFN = Interferon; RAF = Rapidly Accelerated Fibrosarcoma onkogene; *NRAS* = Neuroblastoma RAS viral oncogene homolog; HDAC = Histone Deacetylase; IL = Interleukin; ICI = Immune checkpoint inhibitor

**References:**

1. Wolchok JD, Chiarion-Sileni V, Rutkowski P, Cowey CL, Schadendorf D, Wagstaff J, et al. Final, 10-Year Outcomes with Nivolumab plus Ipilimumab in Advanced Melanoma. N Engl J Med. 2025;392(1):11-22.

2. Schadendorf D, Dummer R, Flaherty KT, Robert C, Arance A, de Groot JWB, et al. COLUMBUS 7-year update: A randomized, open-label, phase III trial of encorafenib plus binimetinib versus vemurafenib or encorafenib in patients with BRAF V600E/K-mutant melanoma. European Journal of Cancer. 2024;204:114073.

3. Robert C, Grob JJ, Stroyakovskiy D, Karaszewska B, Hauschild A, Levchenko E, et al. Five-Year Outcomes with Dabrafenib plus Trametinib in Metastatic Melanoma. N Engl J Med. 2019;381(7):626-36.

4. Tawbi HA, Hodi FS, Lipson EJ, Schadendorf D, Ascierto PA, Matamala L, et al. Three-Year Overall Survival With Nivolumab Plus Relatlimab in Advanced Melanoma From RELATIVITY-047. J Clin Oncol. 2025;43(13):1546-52.

5. Long GV, Hauschild A, Santinami M, Kirkwood JM, Atkinson V, Mandala M, et al. Final Results for Adjuvant Dabrafenib plus Trametinib in Stage III Melanoma. N Engl J Med. 2024;391(18):1709-20.

6. Larkin J, Del Vecchio M, Mandalá M, Gogas H, Arance Fernandez AM, Dalle S, et al. Adjuvant Nivolumab versus Ipilimumab in Resected Stage III/IV Melanoma: 5-Year Efficacy and Biomarker Results from CheckMate 238. Clin Cancer Res. 2023;29(17):3352-61.

7. Eggermont AM, Kicinski M, Blank CU, Mandala M, Long GV, Atkinson V, et al. Seven-year analysis of adjuvant pembrolizumab versus placebo in stage III melanoma in the EORTC1325 / KEYNOTE-054 trial. Eur J Cancer. 2024;211:114327.

8. Luke JJ, Ascierto PA, Khattak MA, de la Cruz Merino L, Del Vecchio M, Rutkowski P, et al. Pembrolizumab Versus Placebo as Adjuvant Therapy in Resected Stage IIB or IIC Melanoma: Final Analysis of Distant Metastasis-Free Survival in the Phase III KEYNOTE-716 Study. J Clin Oncol. 2024;42(14):1619-24.

9. Kirkwood J, Del Vecchio M, Weber J, Hoeller C, Grob JJ, Mohr P, et al. Adjuvant nivolumab in resected stage IIB/C melanoma: primary results from the randomized, phase 3 CheckMate 76K trial. Nat Med. 2023;29(11):2835-43.

10. Blank CU, Lucas MW, Scolyer RA, van de Wiel BA, Menzies AM, Lopez-Yurda M, et al. Neoadjuvant Nivolumab and Ipilimumab in Resectable Stage III Melanoma. N Engl J Med. 2024;391(18):1696-708.

11. Patel SP, Othus M, Chen Y, Wright GP, Yost KJ, Hyngstrom JR, et al. Neoadjuvant-Adjuvant or Adjuvant-Only Pembrolizumab in Advanced Melanoma. N Engl J Med. 2023;388(9):813-23.

12. Long GV, Nair N, Marbach D, Scolyer RA, Wilson S, Cotting D, et al. Neoadjuvant PD-1 and LAG-3-targeting bispecific antibody and other immune checkpoint inhibitor combinations in resectable melanoma: the randomized phase 1b/2 Morpheus-Melanoma trial. Nature Medicine. 2025;31(11):3700-12.

13. Lin CC, Curigliano G, Santoro A, Kim DW, Tai D, Hodi FS, et al. Sabatolimab in combination with spartalizumab in patients with non-small cell lung cancer or melanoma who received prior treatment with anti-PD-1/PD-L1 therapy: a phase 2 multicentre study. BMJ Open. 2024;14(8):e079132.

14. Davar D, Eroglu Z, Pérez CL, Di Pace B, Wang T, Yanamandra N, et al. Combined Targeting of PD-1 and TIM-3 in Patients with Locally Advanced or Metastatic Melanoma: AMBER Cohorts 1c, 1e, and 2A. Clin Cancer Res. 2025;31(16):3433-42.

15. Ascierto PA, Grabbe S, Guida M, Carnevale Schianca F, Rutkowski P, Arance Fernandez AM, et al. 1605MO Primary results from a randomized phase II trial of BNT111 in combination with cemiplimab with calibrator monotherapy arms in anti-PD-(L)1 relapsed/refractory melanoma. Annals of Oncology. 2025;36:S949-S50.

16. Sullivan RJ, Hassel JC, Gebhardt C, Amaral TMS, Grabbe S, Ansstas G, et al. 954P A randomized phase II study of autogene cevumeran plus pembrolizumab (pembro) versus pembro in 1L advanced melanoma (IMcode001). Annals of Oncology. 2025;36:S623-S4.

17. Hassel JC, Arance AM, Carlino MS, Ascierto PA, Sandhu SK, Puzanov I, et al. LBA53 IO102-IO103 cancer vaccine plus pembrolizumab for first-line (1L) advanced melanoma: Primary phase III results (IOB-013/KN-D18). Annals of Oncology. 2025;36:S1712-S3.

18. Rohrberg KS, Garralda E, Calvo E, Moreno Garcia V, Guidi M, Kraus DG, et al. 745P Clinical activity, safety, and PK/PD from the first in human study (NP41300) of RO7247669, a PD1-LAG3 bispecific antibody. Annals of Oncology. 2022;33:S884-S5.

19. Wang Y, Li Y, Liu J, Luo S-x, Li Q, Zou W, et al. SI-B003 (PD-1/CTLA-4) in patients with advanced solid tumors: A phase I study. Journal of Clinical Oncology. 2023;41(16_suppl):e14668-e.

20. Medina T, Chesney JA, Kluger HM, Hamid O, Whitman ED, Cusnir M, et al. Long-Term Efficacy and Safety of Lifileucel Tumor-Infiltrating Lymphocyte Cell Therapy in Patients With Advanced Melanoma: A 5-Year Analysis of the C-144-01 Study. J Clin Oncol. 2025;43(33):3565-72.

21. Rohaan MW, Borch TH, van den Berg JH, Met Ö, Kessels R, Geukes Foppen MH, et al. Tumor-Infiltrating Lymphocyte Therapy or Ipilimumab in Advanced Melanoma. N Engl J Med. 2022;387(23):2113-25.

22. Kähler KC, Hassel JC, Ziemer M, Rutkowski P, Meier F, Flatz L, et al. Neoadjuvant intralesional targeted immunocytokines (daromun) in stage III melanoma. Annals of Oncology. 2025;36(10):1166-77.

23. Wong MK, Milhem MM, Sacco JJ, Michels J, In GK, Muñoz Couselo E, et al. RP1 Combined With Nivolumab in Advanced Anti–PD-1–Failed Melanoma (IGNYTE). Journal of Clinical Oncology. 2025;43(33):3589-99.

24. Shaw HM, Patel PM, Payne M, Kumar S, Danson S, Highley M, et al. A DNA plasmid melanoma cancer vaccine, SCIB1, combined with nivolumab + ipilimumab in patients with advanced unresectable melanoma: Efficacy and safety results from the open-label phase 2 SCOPE trial. Journal of Clinical Oncology. 2024;42(16_suppl):9535-.

25. Bechter O, Loquai C, Champiat S, Baurain JF, Grob JJ, Utikal J, et al. A Phase I, First-in-Human, Dose-Escalation, Expansion Trial of Cytokine-Encoding Synthetic mRNA Mixture Alone or with Cemiplimab in Advanced Solid Tumors. Clin Cancer Res. 2025;31(12):2358-69.

26. Kim U, McCormick TS, Mangla A, Cooper KD, Schwartz GK, Yoshida A. Advances in Cutaneous Melanoma Therapy: The Emerging Role of CDK4/6 Inhibitors. Pharmacol Res. 2025;221:107955.

27. Lelliott EJ, Sheppard KE, McArthur GA. Harnessing the immunotherapeutic potential of CDK4/6 inhibitors in melanoma: is timing everything? NPJ Precis Oncol. 2022;6(1):26.

28. Wang Y, Xu G, Xia H. Targeting the MAPK pathway for NRAS mutant melanoma: from mechanism to clinic. Br J Dermatol. 2025;193(3):381-93.

29. Calvo E, Boni V, Dumas O, Shin SJ, Rosen SD, Chaudhry A, et al. Nemvaleukin alfa monotherapy in patients with advanced melanoma and renal cell carcinoma: results from the phase 1/2 non-randomized ARTISTRY-1 trial. J Immunother Cancer. 2025;13(8):e010777.

30. Ny L, Jespersen H, Karlsson J, Alsén S, Filges S, All-Eriksson C, et al. The PEMDAC phase 2 study of pembrolizumab and entinostat in patients with metastatic uveal melanoma. Nat Commun. 2021;12(1):5155.

31. Khushalani NI, Pereira RP, Nakakogue TD, Segalla J, Liutti VT, Melo Cruz FJS, et al. 1630P HBI-8000 and nivolumab combination in advanced melanoma patients with brain metastases: Analysis of HBI-8000-303 open-label cohort. Annals of Oncology. 2025;36:S967.
